# The State of Research and Weight of Evidence on the Epigenetic Effects of Bisphenol A

**DOI:** 10.3390/ijms24097951

**Published:** 2023-04-27

**Authors:** Ahmad Besaratinia

**Affiliations:** Department of Population and Public Health Sciences, USC Keck School of Medicine, University of Southern California, M/C 9603, Los Angeles, CA 90033, USA; besarati@med.usc.edu; Tel.: +1-(323)-442-0088; Fax: +1-(323)-865-0103

**Keywords:** *DNA* methylation, endocrine disrupting chemical (EDC), histone modifications, noncoding *RNA*, receptors, reproductive system, transcription factors, transgenerational effects

## Abstract

Bisphenol A (BPA) is a high-production-volume chemical with numerous industrial and consumer applications. BPA is extensively used in the manufacture of polycarbonate plastics and epoxy resins. The widespread utilities of BPA include its use as internal coating for food and beverage cans, bottles, and food-packaging materials, and as a building block for countless goods of common use. BPA can be released into the environment and enter the human body at any stage during its production, or in the process of manufacture, use, or disposal of materials made from this chemical. While the general population is predominantly exposed to BPA through contaminated food and drinking water, non-dietary exposures through the respiratory system, integumentary system, and vertical transmission, as well as other routes of exposure, also exist. BPA is often classified as an endocrine-disrupting chemical as it can act as a xenoestrogen. Exposure to BPA has been associated with developmental, reproductive, cardiovascular, neurological, metabolic, or immune effects, as well as oncogenic effects. BPA can disrupt the synthesis or clearance of hormones by binding and interfering with biological receptors. BPA can also interact with key transcription factors to modulate regulation of gene expression. Over the past 17 years, an epigenetic mechanism of action for BPA has emerged. This article summarizes the current state of research on the epigenetic effects of BPA by analyzing the findings from various studies in model systems and human populations. It evaluates the weight of evidence on the ability of BPA to alter the epigenome, while also discussing the direction of future research.

## 1. Introduction

**Figure 1:**
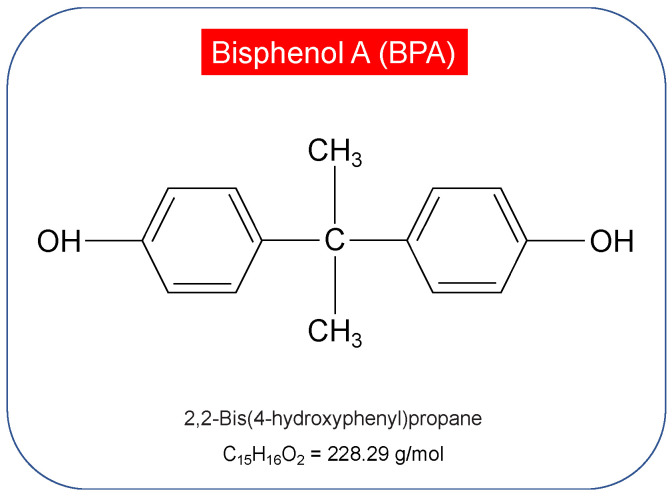
Chemical structure of bisphenol A.

Bisphenol A (BPA) was first synthesized in 1891 by the Russian chemist Aleksandr Pavlovich Dianin; BPA was originally named ‘Dianin’ compound [1]. Dianin’s method for preparing BPA [2,2-Bis (4-hydroxyphenyl) propane, C_15_H_16_O_2_ = 228.29 g/mol] involved catalyzed condensation of a 2:1 mixture of phenol and acetone in the presence of concentrated hydrochloric acid or sulfuric acid [2]. BPA is a colorless, odorless, and solid organic compound, poorly soluble in water, and completely soluble in organic solvents (Figure 1) [3,4]. In the 1950s, it was discovered that reaction of BPA with phosgene (carbonyl chloride, COCl_2_) produces a clear hard resin, known as polycarbonate [2,5]. Following this discovery, BPA has been increasingly used in a myriad of industrial and consumer applications [2,6,7]. BPA is a high-production-volume chemical with numerous utilities [8,9]. BPA is extensively used in the manufacture of polycarbonate plastics and epoxy resins [2,6,7]. Polycarbonate plastics made from BPA have remarkable chemical and physical properties, including excellent strength and rigidity, thermal stability, and resistance to oils and acids [5,6,7]. The epoxy resin of BPA has a viscous consistency, provides strong adhesion and high corrosion resistance, and is commonly used for the coating and inner lining of other products [2,6,7]. BPA monomer is also used in the manufacture of specialty plastics, such as polyester, polysulfone, polyacrylate, and polyetherimide, and as a precursor, developer, additive, or processing aid in the synthesis of other materials [4,8,10,11].

The omnipresence of BPA in consumer products used in day-to-day life is all but inexorable [12,13,14]. Attesting to the versatility and ubiquity of BPA is the widespread use of this chemical as internal coating for food and beverage cans, bottles, and food-packaging materials, and as a building block for goods of common use (e.g., tableware and kitchenware), water pipes, shatterproof windows, impact-resistance safety equipment, toys, storage containers, electronics, computers, compact disks (CDs), digital versatile disks (DVDs), sports equipment, thermal receipts (e.g., cash receipts, movie tickets, and boarding passes), flame retardants, dental fillings or sealants, and medical devices containing polycarbonate or polysulfone plasticizers, such as contact lenses, intravenous cannulas, catheters, probes, inhalers, neonatal incubators, and hemodialysis apparatus [15,16,17,18,19].

It is estimated that more than 13 billion pounds of BPA have entered the global marketplace in 2021 [20]. Emissions from facilities producing BPA or manufacturing BPA-containing materials are substantial [21]; the U.S. Environmental Protection Agency (USEPA) estimates an annual release of over one million pounds of BPA to the environment [22]. BPA can be released into the environment and enter the human body at any stage during its production, or in the process of manufacture, use, or disposal of materials made from this chemical [4,13,23]. For example, during storage, BPA can leach from the protective internal epoxy resin coating of canned foods and bottles and packaging materials into food and beverages [24,25,26,27]. BPA can also be released from polycarbonate plastics or food and drink containers when they are heated in a microwave or washed with harsh detergents [5,13]. Degradation of polymeric materials, such as containers or vessels, is facilitated when they hold saline, acidic, or basic compounds, resulting in the hydrolysis of ester bonds that link BPA monomers [28,29]. Additionally, BPA can leach from consumer products into surface water and soil [13,29,30,31]. BPA can also migrate into dust, e.g., from laminate flooring or paints, by attaching to the solid particulates present in the air [32,33]. Other sources of BPA in the environment include leachates from landfills, discharges of effluents containing BPA from municipal wastewater treatment plants, and combustion of residential waste [31,34,35]. BPA half-life is approximately 4.5 days in water and soil, and less than one day in the air because of its low volatility [4,29].

Most regulatory agencies monitor and regulate BPA exposure in humans based on dietary exposure and aggregate exposures from water, soil, and air [4,8]. In recent years, BPA has been added to the list of banned substances in several consumer products, such as infant feeding bottles (baby bottles), spill-proof cups (sippy cups), infant formula packaging, and cosmetics [36]. In the United States, the use of BPA-containing epoxy resins as coatings for canned foods has recently decreased, although U.S. manufacturers have not abandoned the use of this chemical for other applications, including production of countless variety of polycarbonate consumer goods [37].

## 2. Human Exposure to BPA

While the general population is predominantly exposed to BPA through contaminated food and drinking water, additional exposures from ingestion of dust, inhalation of indoor and outdoor air, and dermal contact or absorption through the eye also occur [38,39,40,41]. In humans, BPA is detectable in various body fluids, such as blood, urine, saliva, sweat, breast milk, and amniotic fluid, as well as on the skin [38,42,43,44,45,46]. BPA can cross the blood–brain barrier and the placenta [5,45,47]. Detectable levels of BPA have been found in human maternal and fetal serum and the human placenta [5,47]. BPA can also accumulate in human tissues, primarily adipose tissue, owing to its lipophilic property (logP = 3.4) [35,48,49]. The widespread presence of BPA in the human body suggests that not only dietary exposure but also non-dietary exposures through the respiratory system, integumentary system (eye and skin contact), and vertical transmission (maternofetal), as well as other routes of exposure, can have significant toxicological relevance given the toxicokinetics of this compound [40,50,51,52].

Upon entering the human body, BPA is rapidly absorbed, distributed, metabolized, and then eliminated mostly through urinary excretion [38,40,41,53,54]. The absorbed BPA is metabolized in the liver through glucuronidation or sulfonation [38,54,55,56,57,58,59], although oxidative metabolism by cytochrome P450 (CYP) enzymes and peroxidases can also occur [60,61,62]. The latter leads to the formation of electrophilic or reactive species, or estrogenic metabolites [5,60,63,64,65,66,67,68] that may bind macromolecules, such as DNA or proteins [63,69,70,71,72,73,74,75]. Conjugation of BPA is mainly catalyzed by the liver enzyme UDP-glucuronosyltransferases 2B15 (UGT2B15) [53,76,77], followed by its excretion from the body via urine [42,51,59]. The half-life of orally absorbed BPA is less than 6 h [54,57,78]. Despite the rapid elimination of BPA from the body, over 90% of urine samples from the studied human populations show detectable levels of this chemical and/or its metabolites [79,80,81]. This finding supports that BPA exposure in humans is constant, recurring, and likely from multiple sources [37,82,83,84]. In confirmation, varying concentrations of BPA or its metabolites can be found in human urine or other body fluids over time or at different intervals within a short span of time, e.g., a single day [83,85].

The temporal changes in BPA levels in human tissues or matrices pose a major challenge for biomonitoring studies because a single measurement of BPA, e.g., in spot urine samples, can only provide information on recent, but not long-term exposure to this chemical [83,85,86]. This may explain why in occupational studies, estimates of long-term exposure to BPA, based on questionnaire data or job exposure matrices, do not correlate well with urinary BPA levels measured in the study participants [4]. Further jeopardizing the accuracy and reliability of BPA measurement in human specimens are problems relating to the external contamination of supplies, consumables, and instruments needed for sampling and/or analytical processing [87,88,89]. To date, accurate and reliable quantification of human exposure to BPA, particularly long-term exposure, remains a formidable task for population-based studies.

## 3. Biological Effects of BPA

The first evidence on BPA’s ability to exert biological effects was obtained in 1936 by Dowds and Lawson, who discovered the estrogenic properties of this chemical in vivo [90]. In 1997, an estrogen-receptor-dependent mechanism of action for BPA was elucidated that involved estrogen receptors ERα and ERβ [3,91,92]. Owing to its structural similarity to estradiol (i.e., major female sex hormone), BPA can interfere with steroid signaling, thereby causing reproductive health outcomes, depending on the window of exposure, dosage, duration and mode of exposure, and developmental life stage [3,93,94,95,96]. The European Chemicals Agency has classified BPA as a reproductive toxicant and a substance of very high concern [97]. The National Toxicology Program (NTP) Center for the Evaluation of Risks to Human Reproduction has stated, “The NTP has some concern for effects on the brain, behavior, and prostate gland in fetuses, infants, and children at current human exposures to bisphenol A” [8,22,98].

BPA is often classified as an endocrine-disrupting chemical (EDC) as it can act as a xenoestrogen [5,8,99]. EDCs can mimic or antagonize endogenous hormones by interfering with their synthesis or clearance, resulting in developmental, reproductive, cardiovascular, neurological, or immune effects, metabolic disorders, or oncogenesis in both humans and animals [35,100,101,102,103,104,105,106]. Importantly, the endocrine system is most vulnerable to assaults by EDCs during the prenatal and early development window, and the induced effects may persist into adulthood and be passed on to future generations [107,108,109,110,111]. Growing evidence shows that not only EDCs can directly affect various organ systems in humans and animals, but they can also exert transgenerational effects, presumably through placental exposure in fetus or lactational exposure in offspring [99,108,111].

BPA disrupts the synthesis, secretion, release, and transport of hormones by interacting with biological receptors, such as the androgen receptor (AR), thyroid hormone receptor (THR), estrogen-related receptor gamma (ERRγ), and glucocorticoid receptor (GR), as well as other nuclear and membrane estrogen receptors (ERs), such as G-protein-coupled estrogen receptor (GPER/GPR30) and estrogen-related receptor γ (ERRγ), and other nuclear receptors, e.g., constitutive androstane receptor (CAR), glucocorticoid receptor (GR), liver X receptor (LXR), peroxisome proliferator-activated receptor β/δ (PPARβ/δ), retinoic acid receptor (RAR), and retinoid X receptor (RXR) [100,112,113,114,115,116,117,118]. For example, BPA exhibits estrogenic, antiestrogenic, and antiandrogenic activities at multiple levels along the hypothalamus–pituitary–gonad (HPG) axis, which is a main regulator of reproductive system [95,119].

An alternative mode of action for BPA is its interaction with key transcription factors (TFs) to modulate regulation of gene expression (reviewed in [4,119,120,121]). Accumulating data suggest that BPA interacts with adipogenic TFs, such as peroxisome proliferator-activated receptors (PPARs), CCAAT-enhancer-binding proteins (C/EBPs), and nuclear factor erythroid 2-related factor 2 (Nrf2), to exert obesogenic effects [119,120,121]. In addition, TFs from homeobox gene (HOX) family and heart- and neural crest derivatives-expressed protein 2 (HAND2) are thought to play a crucial role in BPA-mediated detrimental effects [4,119,121].

**Figure 2:**
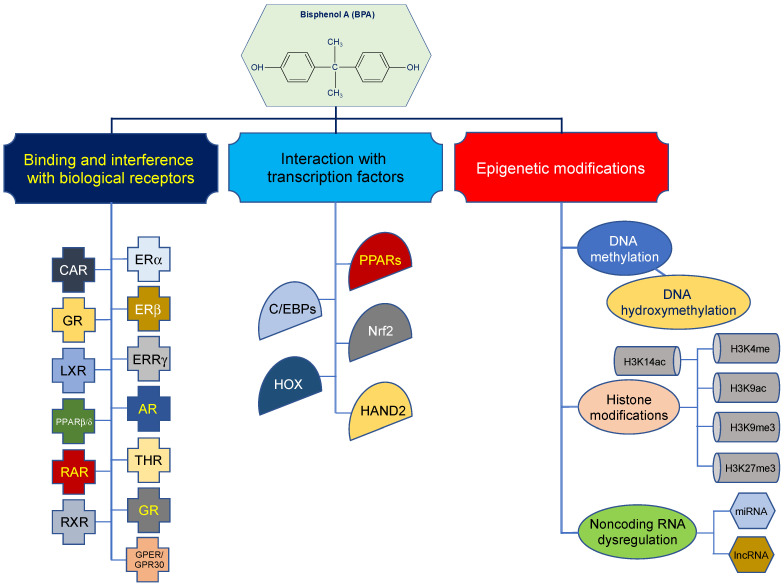
Molecular mechanisms of action of bisphenol A. The mechanisms of action of BPA are depicted, including (1) binding and interference with biological receptors, (2) interaction with transcription factors, and (3) epigenetic modifications. For brevity, the most investigated biological receptors, transcription factors, and histone modifications are shown. The figure is a simplified summary but not an exhaustive delineation of the molecular mechanisms of bisphenol A. AR = androgen receptor; CAR = constitutive androstane receptor; C/EBPs = CCAAT-enhancer-binding proteins; ERs = estrogen receptors; ERRγ = estrogen-related receptor γ; GPER/GPR30 = G-protein coupled estrogen receptor; GR = glucocorticoid receptor; H3K4me = histone 3 lysine 4 methylation; H3K9ac = histone 3 lysine 9 acetylation; H3K9me3 = histone 3 lysine 9 trimethylation; H3K27me3 = histone 3 lysine 27 trimethylation; H3K14ac = histone 3 lysine 14 acetylation; HAND2 = heart- and neural crest derivatives-expressed protein 2; HOX = homeobox gene family; LXR = liver X receptor; Nrf2 = nuclear factor erythroid 2-related factor 2; PARs = peroxisome proliferator-activated receptors; PPARβ/δ = peroxisome proliferator-activated receptor β/δ; RAR = retinoic acid receptor; RXR = retinoid X receptor; THR = thyroid hormone receptor.

A third mechanism of action for BPA has emerged that involves epigenetic modifications (Figure 2) [106,122,123]. In the following section, the *‘knowns’* and *‘unknowns’* of the epigenetic effects of BPA are discussed. The current state of research on the effects of BPA on the epigenome is summarized, weight of evidence on the ability of BPA to induce epigenetic modifications is appraised, and direction of future research is outlined. Interested readers are referred to comprehensive and updated reviews on the first two mechanisms of action of BPA, including (I) binding and interference with biological receptors and (II) interaction with TFs [4,119,120,121].

## 4. Epigenetic Effects of BPA

As of 20 March 2023, a PubMed search with the terms “*bisphenol A*” and “*epigenetics*” has yielded 384 publications, of which 130 are ‘Review’ articles. The sheer number of publications on this topic is impressive, considering that investigating the epigenetic effects of BPA started around 2006. Ho et al. [124] were first to demonstrate that neonatal exposure of rats to low, environmentally relevant doses of BPA led to hypomethylation of CpG islands in multiple genes, of which one gene (phosphodiesterase 4D (PDE4D4)) showed concomitant transcriptional silencing. The induced epigenetic effects in the exposed animals were associated with increased susceptibility of the prostate gland to adult-onset precancerous lesions and hormonal carcinogenesis [124]. A year later, Dolinoy et al. [125] showed that prenatal exposure of Agouti mice to BPA resulted in a shift in coat color in the offspring; the coat color shift was due to hypomethylation in nine CpGs within an intracisternal A particle (IAP) retrotransposon upstream of the agouti gene. The hypomethylating effect of BPA in the Agouti mice was reversed by maternal dietary supplementation with folic acid, a methyl group donor [126]. Reversal of the BPA-induced hypomethylation was manifested as the restoration of coat color in the offspring [125].

Following the publication of these two seminal reports [124,125], there has been a flurry of research into the epigenetic effects of BPA and other EDCs [52,106,120,122,123,127,128]. So far, three distinct, yet inter-related, types of epigenetic modification [129,130,131,132] have been investigated in relation to BPA exposure. These include aberrant *DNA* methylation, histone modifications, and noncoding *RNA* dysregulation [132,133,134,135]. Of the three, aberrant *DNA* methylation is the most extensively studied epigenetic alteration [129,132]. Relatedly, studies on *DNA* hydroxymethylation and BPA exposure are also beginning to emerge [136,137,138,139,140,141,142]. Collectively, studies on the epigenetic effects of BPA have been performed in cell cultures (in vitro), experimental animals (in vivo), and human populations. The in vitro studies have utilized various cell types, both primary cells and cancer cell lines, generated from human and rodent tissues, and less frequently sheep and fish tissues [143,144,145]. The in vivo studies have used different model systems, such as mice, rats, gerbils, and fish [109,138,146,147,148,149,150,151,152,153,154,155,156,157]. The human studies have largely focused on BPA-exposed individuals, drawn from the general population or occupational settings [52,106,120,127]. Interested readers can find brief summaries of the published studies on the epigenetic effects of BPA in Tables G2–G6 in ref. [4].

As for *DNA* methylation studies, a large number of in vitro [4,139,158,159,160,161,162,163,164,165,166,167,168,169,170,171,172] and in vivo experiments [4,120,173,174] and many human population studies [52,106,127,146,175] have examined the association between aberrant *DNA* methylation and BPA exposure. Based on the in vitro data, there is evidence that exposure to BPA is associated with gain or loss of *DNA* methylation (hyper- or hypomethylation, respectively) in single genes or in gene panels [4,122]. The observed associations have mostly been cell-type dependent, as shown in cell culture experiments, whereby a wide variety of cell types from different species were treated with BPA at varying doses (mostly in the nanomolar to micromolar range) [164,174,176]. Following the treatment, some, but not all, of the examined cell types have shown methylation changes in candidate genes, e.g., in the promoter region of single genes or in repeat elements [160,164,177]. The methylation changes have been detectable in cells treated at some, but not all, doses of BPA. As such, establishment of a dose-response relationship between BPA exposure and aberrant *DNA* methylation has not been straightforward [160,164,177]. Pathway analysis of the differentially methylated genes has been performed in a few in vitro studies. The results have shown enrichment of molecular pathways implicated in cancer, neurodevelopment, metabolism, and reproduction, among others [168].

Similar findings have been reported in animal studies wherein direct administration of BPA or its transplacental and translactational exposures have been associated with aberrant *DNA* methylation in individual genes or gene panels in rodents and fish or in embryos, neonates, or offspring (both juveniles and adults) of the exposed animals [178,179,180,181,182,183,184,185,186,187,188,189,190,191,192,193,194,195]. The methylation changes associated with BPA exposure have been tissue- or sex-specific in some of the conducted studies [138,182,185,196]. Furthermore, a few in vivo studies have evaluated genome-wide *DNA* methylation changes in the liver, uterine, or mammary gland tissues of mouse or rat offspring, exposed perinatally to BPA (i.e., through maternal diet or by intraperitoneal injection of BPA to dams or via oral gavage) [4,106]. The analyzed tissues have shown differentially methylated regions (DMRs), which were mostly enriched in pathways involved in cancer, signaling, stimulus response, and metabolism [184]. Other in vivo studies and some in vitro experiments have measured the expression of enzymes that catalyze *DNA* methylation [197,198], including *DNA* methyltransferases (DNMTs), specifically DNMT1, DNMT3A, and DNMT3B [136,141,162,167,168,172,176,179,180,182,183,194,195,199,200,201]. Both over-expression and under-expression of the maintenance and de novo DNMTs have been observed in in vivo and in vitro experiments. The relationship between DNMT levels and methylation status in the tested genes has not, however, been direct, as can be expected [4,106,197,198,202].

Human studies on BPA exposure and aberrant *DNA* methylation have mostly been conducted in maternally exposed individuals (mother–child pairs) [52,106,193,203] or subjects with environmental or occupational exposure to this chemical [204,205,206,207,208,209]. The vast majority of these studies have investigated *DNA* methylation status in candidate genes or gene panels in relation to BPA exposure [52,106,120,127]. Associations between aberrant *DNA* methylation and BPA exposure have been found in some of these studies, although there have also been divergent results [52,106,120,127]. Human studies have also examined the relationship between BPA exposure and *DNA* methylation status at a global level [52,106,120,127]. With a few exceptions, however, the global effects of BPA on *DNA* methylation in humans have not been truly investigated in a genome-wide fashion [175,210,211,212]. In fact, investigations of global *DNA* methylation changes in humans exposed to BPA (e.g., in maternally, environmentally, or occupationally exposed individuals) have either used methylation arrays, which interrogate only a fraction of the CpGs of the genome, or use antibody-based assays or other enrichment methods to analyze certain repeat elements (e.g., long- or short-interspersed nuclear elements (LINEs or SINEs, resp.) or satellite repeats as a proxy for the CpG content of the entire genome [47,205,213,214]. For instance, earlier studies have used the GoldenGate arrays (Illumina, Inc., San Diego, CA, USA) that screen only about 1500 CpGs [215]. More recently, 27 k or 450 k Infinium arrays (Illumina, Inc., San Diego, CA, USA) have also been used [193,211,212]. The latter arrays enable interrogation of <2% of the CpGs of the whole genome [211,212]. Of note, the human genome consists of more than 28 million CpGs [216,217]. Thus, there is a need to study the effects of BPA on the whole methylome, using high throughput, scalable, and fast techniques, such as next-generation sequencing-based platforms. Considering the complexity of human exposure to BPA, the results of *DNA* methylation analysis in human populations need to be carefully examined and cautiously interpreted (discussed in ‘Section 5’).

To a much lesser extent, studies on two other types of epigenetic changes, including histone modifications and noncoding *RNAs* dysregulation, have also been conducted, although primarily in cell culture systems and animal models [106,120,218,219]. The histone modification studies have focused on representative active or repressive histone marks (e.g., H3K4me, H3K9ac or H3K9me3, and H3K27me3), also measuring enzymes that catalyze these reactions, including histone acetyltransferases (HATs), histone methyltransferases (HMTs), histone deacetylases (HDACs), and histone demethylases (KDMs), and quantifying the expression of associated genes [139,164,167,186,196,220,221,222,223,224,225,226,227,228,229,230,231,232,233,234,235,236]. For instance, BPA treatment of human breast cancer cells (MCF7) resulted in increased histone acetylation and H3K4 trimethylation through enrichment of the mixed-lineage leukemia family of histone methyltransferases (MLL2 and MLL3) and CREB-binding protein and p300 (CBP/p300; paralogous lysine acetyltransferases) at the promoter of HOXC6, HOXB9, and the enhancer of Zeste homolog 2 (EZH2) that are involved in breast cancer and other types of cancer [222,223,227]. Furthermore, primary human endometrial cells and several other cancer cell lines treated with BPA showed significant changes in global histone acetylation and methylation (H3K9ac, H3K9me3, H3K4me3, H3K18/23 diacetylation, H3K27me3, or H4K20me3) [139,164,232,233,235]. In addition, BPA treatment significantly changed the expression levels of various HDACs, including HDAC1, HDAC3, HDAC5, and HDAC7, and HATs in both normal and cancer cell lines [139,232,233,235]. In vivo treatment of rats, mice, and gerbils with BPA resulted in elevated expression of histone methyltransferase EZH2 [157,220,222,228]. Additionally, BPA treatment led to significant changes in histone marks (H3Ac, H4Ac, H3Me2K4, H3K9me, H3K27me3, or H3Me3K36) in rat liver [196], pancreas [237], mammary gland [228,238], and testes [239] and in mouse mammary gland [220] and testes [225,231]. Moreover, zebrafish and rare minnows exposed to BPA showed altered histone methylation or acetylation and changes in expression levels of HDACs in liver and testicular cells, testes, ovaries, embryos, and larvae [109,145,149,153,154,155,234,240,241].

Likewise, dysregulation of noncoding *RNAs*, specifically *microRNAs* (*miRNAs*) [171,242,243,244,245,246,247,248,249,250,251,252,253,254,255,256] and long noncoding *RNAs* (*lncRNAs*) [143,144,248,257,258], has been investigated in in vitro and in vivo experiments and human studies. In vitro treatment of human primary adipocytes, lung fibroblasts, and other cancer cell lines (endometrial, breast, and colon cancer cells) with BPA resulted in altered expression of many noncoding *RNAs*, including up- and downregulation of several dozen *miRNAs* and *lncRNAs* [171,243,247,248,253]. The dysregulated noncoding *RNAs* in the BPA-treated cells are known to modulate key biological processes, including cell cycle, metabolism, stimulus response, and inflammation, among others. Furthermore, in utero exposure of mice to BPA led to significant changes in expression of *miRNAs*, including anti-inflammatory miR146 or its isoforms that are key mediators of neurobehavioral disorders and metabolic changes [254]. De Felice et al. [246] have reported that miR-146a was significantly overexpressed and correlated with BPA accumulation in the placenta from pregnant women who lived in a polluted area in southern Italy and underwent therapeutic abortion because of fetal malformations. Kim et al. [252] investigated the relationship among BPA exposure, miRNA expression, and hypertension (high blood pressure) in a randomized crossover trial with 45 nonsmoking females over 60 years of age. Using the mixed-effects models, the authors demonstrated that decreases in miR-30a-5p, miR-580-3p, miR-627-5p, and miR-671-3p and increases in miR-636 and miR-1224-3p attributable to BPA exposure were associated with hypertension. The core functional network from BPA exposure to hypertension was found to be on the pathway through these six *miRNAs* and their predicted hypertension-related target genes. Palak et al. [255] have shown significantly higher levels of BPA together with upregulation of miR-let-7a, miR-let-7b, and miR-let-7c and downregulation of miR-518f in the seminal plasma of azoospermic men as compared to healthy controls (azoospermia: complete absence of spermatozoa in semen specimens). Whereas BPA levels were positively correlated to miR-let-7a and miR-let-7c levels, there was a negative correlation between BPA and miR-518f levels in seminal plasma. The levels of BPA in seminal plasma were also negatively correlated to sperm concentration and normal semen morphology.

Altogether, there is evidence that in vitro and in vivo exposures to BPA are associated with histone modifications, as well as with upregulation or downregulation of diverse *miRNAs* or *lncRNAs*, many of which are implicated in pathogenic pathways involved in various diseases, such as cancer, reproductive, neurobehavioral, cardiovascular, and metabolic diseases, and inflammation [171,242,257]. Notwithstanding, the findings of human studies on dysregulation of noncoding *RNAs* in relation to BPA exposure need to be interpreted cautiously (discussed in ‘Section 5’).

## 5. Limitations of Research on the Epigenetic Effects of BPA in Humans

As discussed in the preceding section, BPA exposure has been associated with epigenetic changes, mainly aberrant *DNA* methylation, in a number of studies in human populations [52,106,120,127]. It is prudent, however, to carefully examine the findings of these studies and interpret their results cautiously. A main concern for epigenomic studies in human populations is the epigenetic plasticity [259]. The human epigenome changes dynamically according to physiologic state and pathologic conditions [129,130,131,132]. This is represented by the continuous shaping and reshaping of the epigenome during the developmental stage, aging, or consequent to exposure to a wide variety of chemical or physical agents attributable to lifestyle factors (e.g., smoking), occupation, medical treatments, diet, and environment, as well as various diseases and conditions [129,260,261,262,263]. Thus, associating epigenetic changes to any given exposure in human populations is tremendously challenging. Epigenomic studies in diverse human populations that do not satisfactorily account for various determinants of the epigenetic plasticity are all but certain to introduce biases into the results. In the case of BPA, this situation might be even more complicated, considering the pervasiveness of this chemical in the environment and the constant, recurring, and multiple-source exposure of humans to this compound. This is further compounded by the toxicokinetics of BPA and lack of long-term exposure biomarkers for this chemical [37,82,83,84].

The above complications are likely to have impacted many mother–child pair studies in which association between BPA exposure and aberrant *DNA* methylation has been investigated. In most of those studies, spot urine samples from mothers were collected for BPA measurement to find its association with *DNA* methylation in fetal tissues, placenta, or cord blood, or in peripheral blood from offspring 2 to 14 years after birth [193,203,214,264,265,266,267,268]. The accuracy and representativeness of a one-time measurement of BPA in mothers’ urine is, at best, non-optimal when used for estimating the gestational exposure of fetus or the exposure of newborns years after birth. This is further complicated by the continued exposure of the newborns to other sources of BPA and the influence of other modulators of the epigenome as the newborns grow. Similar concerns also apply to *DNA* methylation analysis in relation to BPA exposure in boys and girls or adults whose urine or serum samples were taken at a single time or twice a year to make an average for annual BPA exposure [204,206].

A further concern for methylome analysis in relation to BPA exposure in humans is the use of heterogenous tissues or mixed cells despite the fact that epigenetic marks are mostly cell-type specific, as demonstrated in in vitro and in vivo studies (see, ‘Section 4’). For instance, the use of whole blood or placenta in human studies may constitute a limitation [47,208,212,215]. Considering that blood is comprised of various cell types, methylation changes associated with BPA exposure can simply be caused by changes in blood cell composition because of exposure to BPA or other chemicals. Another concern for epigenomic studies in individuals occupationally exposed to BPA is the use of less-than-optimal biospecimens. For example, *‘pooled’* sperm samples from 30 workers in a factory manufacturing BPA and 26 controls were used for hydroxymethylated *DNA* immunoprecipitation sequencing (5-hMeDIP-seq) [137]. A further limitation is the use of potentially compromised study subjects in several human studies. For instance, women receiving reproductive medications and undergoing in vitro fertilization (IVF) were the study subjects for an investigation in which association between BPA levels in serum and *DNA* methylation changes in whole peripheral blood has been studied [215]. One cannot rule out the possibility that methylation changes found in the study subjects were due to administration of the fertility drugs or use of therapeutics [215] and not exposure to BPA.

## 6. Concluding Remarks

One may argue that drawing firm conclusions from the results of published studies on the epigenetic effects of BPA in humans is challenging, considering the design of the conducted studies and the quality of the available data. It is, therefore, reasonable to contemplate conducting future studies that are better designed and sufficiently powered and include well-characterized populations whose exposure to BPA can be accurately and reliably assessed. This would not, however, be an easy undertaking, given the complex nature of human exposure to BPA, lack of long-term exposure biomarkers for this chemical [37,82,83,84], and the significant costs, time, and efforts that would be needed to carry out such studies. Added to these complexities is the long wait time that will be needed to obtain results from any such studies. A complementary approach would be to conduct more comprehensive mechanistic studies in relevant in vitro and in vivo model systems to investigate how BPA exposure may functionally alter the epigenome to cause specific diseases. In this regard, one must be cognizant of the limitations of in vitro and in vivo models and the issues regarding their comparability to humans [269,270,271,272,273,274,275,276]. A great advantage for such follow-up studies is the possibility of leveraging banked specimens from many of the published in vitro and in vivo experiments. This would save significant time, effort, and resources that would otherwise be needed to start any new studies. An important task for future studies is to explore the functional consequences of BPA-associated epigenetic modifications, using integrative multi-omics approaches. This is an important area with great translational potential, which remains highly understudied. The federally funded Consortium Linking Academic and Regulatory Insights on BPA Toxicity (CLARITY-BPA) program [277] and the Toxicant Exposures and Responses by Genomic and Epigenomic Regulators of Transcription (TaRGET) program [278] are two invaluable resources, whereby investigators can maximize the use of existing biospecimens and data for future studies.

## Data Availability

All data are contained within the article.

## Abbreviations

5-hMeDIP-seq, hydroxymethylated DNA immunoprecipitation sequencing; AR, androgen receptor; BPA; bisphenol A; CAR; constitutive androstane receptor; CBP/p300; CREB-binding protein and p300; CD, compact disk; C/EBPs, CCAAT-enhancer-binding proteins; CLARITY-BPA, Consortium Linking Academic and Regulatory Insights on BPA Toxicity; DMRS, differentially methylated regions; DNMTs, DNA methyltransferases; DVD, digital versatile disk; EDC, endocrine-disrupting chemical; EPA, United States Environmental Protection Agency; ER, estrogen receptor; ERRγ, estrogen-related receptor γ; EZH2; enhancer of Zeste homolog 2; GPER/GPR30; G-protein coupled estrogen receptor; GR, glucocorticoid receptor; HAND2, heart- and neural crest derivatives-expressed protein 2; H3K4me; histone 3 lysine 4 methylation; H3K9ac; histone 3 lysine 9 acetylation; H3K9me3; histone 3 lysine 9 trimethylation; H3K14ac; histone 3 lysine 14 acetylation; H3K27me3; histone 3 lysine 27 trimethylation; HATs, histone acetyltransferases; HDACs, histone deacetylases; HMTs, histone methyltransferases; HOX, homeobox gene; HPG, hypothalamus-pituitary-gonad axis; IAP, intracisternal A particle; IVF, in vitro fertilization; KDMs, histone demethylases; LINEs, long interspersed nuclear elements; lncRNAs, long noncoding RNAs; LXR; liver X receptor; miRNAs, microRNAs; MLL; mixed-lineage leukemia; NTP, National Toxicology Program; Nrf2, nuclear factor erythroid 2-related factor 2; PDE4D4, phosphodiesterase 4D; PPARs, peroxisome proliferator-activated receptors; PPARβ/δ; peroxisome proliferator-activated receptor β/δ; RAR; retinoic acid receptor; RXR = retinoid X receptor; SINEs, short interspersed nuclear elements; TaRGET, Toxicant Exposures and Responses by Genomic and Epigenomic Regulators of Transcription; TF, transcription factor; THR, thyroid hormone receptor; USEPA, United States Environmental Protection Agency; UGT2B15, UDP-glucuronosyltransferases 2B15.
